# *Vibrio metschnikovii*, a Potential Pathogen in Freshwater-Cultured Hybrid Sturgeon

**DOI:** 10.3390/ani12091101

**Published:** 2022-04-24

**Authors:** Zidong Xiao, Xudong Li, Mingyang Xue, Mengwei Zhang, Wei Liu, Yuding Fan, Xihua Chen, Zhipeng Chu, Feilong Gong, Lingbing Zeng, Yong Zhou

**Affiliations:** 1 Yangtze River Fisheries Research Institute, Chinese Academy of Fishery Sciences, Wuhan 430223, China; xiaohzau@163.com (Z.X.); xmy@yfi.ac.cn (M.X.); zhangmengwei1103@126.com (M.Z.); liuwei@yfi.ac.cn (W.L.); fanyd@yfi.ac.cn (Y.F.); chenxh@yfi.ac.cn (X.C.); chuzhipeng@yfi.ac.cn (Z.C.); zlb@yfi.ac.cn (L.Z.); 2 Department of Aquatic Animal Medicine, College of Fisheries, Huazhong Agricultural University, Wuhan 430070, China; 3 Henan Fishery Technical Extension Station, Zhengzhou 450008, China; hnsclxd@163.com; 4 Zhengzhou Fishery Technical Extension Station, Zhengzhou 450006, China; gongfeilong69@163.com

**Keywords:** aquaculture, biochemical identification, histopathology, hematological parameters, drug sensitivity

## Abstract

**Simple Summary:**

In an era of shrinking stocks and intensive fisheries production, pathogenic microorganisms are a serious threat to fish health. This study confirms that *Vibrio metschnikovii*—a pathogen mainly found in aquatic environments—was the pathogenic bacterium causing disease in hybrid sturgeon. The authors reveal the hazard of *V. metschnikovii* to hybrid sturgeon and the potential risks to the sturgeon farming industry.

**Abstract:**

In July 2021, a disease with a high mortality rate broke out in freshwater cultured hybrid sturgeon in Zhengzhou, Henan Province. A dominant strain, H-701, was isolated from diseased fish; physiological changes in diseased fish were investigated and molecular identification, biochemical characterization, and pathogenicity and drug sensitivity tests of H-701 were performed. The 16S rRNA gene sequence of H-701 was 99.86% homologous with that of *Vibrio metschnikovii* in GenBank. The 50% lethal dose of H-701 was 3.72 ± 0.929 × 10^4^ CFU/g fish weight. The proportion of monocytes, neutrophils, and eosinophils in the blood of diseased sturgeon increased significantly, whereas the proportion of lymphocytes decreased. In diseased fish, the serum levels of total protein, albumin, globulin, and alkaline phosphatase decreased significantly, and those of aspartate aminotransferase, alanine aminotransferase, and complement C3 increased significantly. There were obvious pathological changes in several tissues of the diseased fish. H-701 was sensitive to antibiotics such as florfenicol, enrofloxacin, and doxycycline. This study not only demonstrated that *V. metschnikovii* was the cause of death of a large number of hybrid sturgeon but also revealed its potential risk in hybrid sturgeon aquaculture. The results provide a basis for the diagnosis and prevention of this disease.

## 1. Introduction

Sturgeons, belonging to the order Acipenseriformes, are among the most primitive fishes in existence [1]. Sturgeons have great research value and extremely high economic value [2]. Owing to the changes in their living environment and the overexploitation and utilization of their resources, most sturgeon species in nature are classified as endangered [3]. They are protected and managed by the International Union for Conservation of Nature (IUCN; www.iucn.org) and the Convention on International Trade in Endangered Species (CITES) [4]. Considering the lengthy maturation period of sturgeon species under natural conditions, it takes a long time for their populations to recover after being destroyed; however, the reproductive ability of hybrid sturgeon is higher than that of the parents which signifies the advantages of hybridization [5,6]. Hybrid sturgeon species (*Huso dauricus* ♀ × *Acipenser schrenckii* ♂, *Acipenser schrenckii* ♀ × *Huso dauricus* ♂, and *Acipenser baerii* ♀ × *Acipenser schrenckii* ♂) constitute the main commercial breeding varieties in China and are associated with remarkable economic benefits [3].

At present, bacterial and viral diseases are the most important diseases in sturgeon culture. The main bacterial pathogens are *Aeromonas hydrophila* [7], *Streptococcus iniae* [8], *Streptococcus dysgalactiae* [9], *Edwardsiella tarda* [10], and *Mycobacterium ulcerans* [11]. The viral pathogens common in sturgeon are Acipenser Iridovirus European [12], sturgeon nucleocytoplasmatic large DNA virus [13], herpesviruses, and mimiviruses [14]. Bacterial infections are considered one of the main sources of sturgeon disease in China [7,8,9,10].

In July 2021, a large number of hybrid sturgeon died at a sturgeon breeding company in Zhengzhou, Henan Province. Initially, some sick hybrid sturgeon in a pond stopped eating and swam slowly. After 5 days, the diseased hybrid sturgeon began to die, with a gradual increase in mortality, resulting in more than 50 fish deaths per day. The death of the sick fish lasted for 15 days, and the condition was controlled after drug treatment. The present study aimed to investigate the etiology of this disease. To this end, a bacterial strain was isolated from a naturally diseased hybrid sturgeon and identified by phenotypic and molecular analysis, biochemical analysis, and a pathogenicity test. Blood analysis of diseased hybrid sturgeon and histopathological injury observations were performed to investigate the changes in blood parameters and pathological characteristics of the injury.

## 2. Materials and Methods

### 2.1. Fish

The diseased hybrid sturgeon (*Acipenser baerii* ♀ × *Acipenser schrenckii* ♂) (body length approximately 65 ± 10 cm) came from an aquaculture company in Zhengzhou city, Henan Province. We made a preliminary diagnosis of the diseased hybrid sturgeon (*n* = 6) on site, recorded the clinical symptoms, and collected fresh blood and tissue samples. Some of the diseased hybrid sturgeon (*n* = 6) were transported to the laboratory in oxygenated bags to isolate the pathogen. Healthy hybrid sturgeon (*n* = 600) with no disease history (body length approximately 15 ± 2 cm) were purchased from a sturgeon farm in Yichang city, Hubei Province. Healthy hybrid sturgeon were kept in a recirculating aquaculture system for 14 days to allow them to acclimatize to the environment. During the acclimatization period, the water temperature was 20 ± 1 °C and the fish were fed with commercial feed twice a day. All animal experiments were approved by the Animal Experimental Ethical Inspection of Laboratory Animal Centre, Yangtze River Fisheries Research Institute, Chinese Academy of Fishery Sciences (ID Number: YFI 2021-zhouyong-06).

### 2.2. Isolation

Diseased sturgeon were euthanized in 0.04% tricaine methane sulfonate (MS-222; Sigma, Saint Louis, MO, USA). The liver and kidney tissues of diseased sturgeon were sampled with a sterile inoculation loop, streaked on Brain Heart Infusion (BHI; HopeBio, Qingdao, China) agar medium, and cultured at 28 °C for 24 h. Single colonies were selected and streaked on BHI agar to obtain pure culture colonies. Two dominant colonies were picked on each plate, and a total of 12 bacteria were obtained. Picked colonies were streaked on BHI agar to obtain pure cultures. After 16S rRNA gene sequencing of 12 isolates, the isolates were identified as the same species. The isolated strain was named H-701.

### 2.3. Morphological Analysis

The H-701 colony was diluted with phosphate buffer saline (PBS). The diluted solution was applied on glass slides, dried, fixed, and stained with Gram stain (Jiancheng, Nanjing, China), and the morphological characteristics of the cells were observed using an optical microscope (Olympus, Tokyo, Japan) [15]. The bacteria were fixed in 2.5% glutaraldehyde solution and dried by dehydration. The morphology of the bacteria was observed, and images were taken using a scanning electron microscope (Hitachi, Tokyo, Japan) [16].

### 2.4. 16S rRNA Gene Analysis

The H-701 strain DNA was extracted using a Bacterial Genomic Extraction Kit (Tiangen, China). PCR amplification was performed using 16S rRNA gene universal primers (F: 5′-AGAGTTTGATCATGGCTCAG-3′, R: 5′-TACGGTTACCTT GTTACGACTT-3′) [17]. PCR conditions were as follows: 30 s at 94 °C, 30 s at 57 °C, and 90 s at 72 °C for 32 cycles [18]. PCR products were resolved on 1.5% agarose gel electrophoresis. The gel recovery kit (OMEGA, GA, USA) was used for DNA recovery from agarose gels. The DNA was cloned into the pMD19-T vector (TaKaRa, Dalian, China) and sequenced (Huayu Gene, Inc., Wuhan, China).

### 2.5. Biochemical Identification

The biochemical identification was accomplished using a Biolog microbial identification system (Biolog, Hayward, CA, USA). The H-701 strain was picked with a sterile cotton swab and inoculated into the IF-A inoculation solution. The IF-A inoculum solution with strain H-701 was then added to the GEN III assay plate (100 µL per well). The GEN III identification plate was placed into the Biolog microbial identification system for automatic identification.

### 2.6. Blood Parameter Analysis

Differential leukocyte counts (DLC) were performed to classify and count leukocytes. Diseased hybrid sturgeon (*n* = 6) and healthy hybrid sturgeon (*n* = 6) were anesthetized, and blood was drawn from the tail vein using a disposable sterile syringe and immediately added to a slide to make a blood smear. Three blood smears were made per fish and stained with Wright–Giemsa stain (Baso, Zhuhai, China). White blood cells (*n* = 200) were observed on each blood smear under the oil lens of a microscope and classified and counted [19].

For the determination of serum biochemical indicators, the drawn blood was placed in a centrifuge tube to allow natural coagulation, left at 4 °C for 30 min, and centrifuged at 2000× *g* for 10 min. The upper serum layer was transferred to a new centrifuge tube for testing. The activity of aspartate aminotransferase (AST), alanine aminotransferase (ALT), alkaline phosphatase (ALP) and content of total protein (TP), albumin (ALB), globulin (GLB), and complement C3 in the serum were determined by the automatic biochemical analyzer (Sysmex, Kobe, Japan).

### 2.7. Observation of Histopathology

The liver, spleen, kidney, gill, intestine, and heart tissues of diseased hybrid sturgeon were collected, fixed in 4% paraformaldehyde solution for 24 h, subjected to gradient alcohol dehydration, and embedded in paraffin. Continuous sectioning (5 µm) was performed using a rotary microtome (Leica, Wetzlar, Germany); the sections were stained with hematoxylin-eosin (HE, Solarbio, Beijing, China) and sealed with neutral resin [20]. Histopathological changes were observed with an optical microscope (Olympus, Tokyo, Japan).

### 2.8. Pathogenicity

Bacterial concentration was calculated using the plate colony counting method [21]. The healthy hybrid sturgeon were randomly divided into six groups with 30 fish per group. The experimental fish in each group were raised in an independent circulating water system, and the size of the water tank was 1 m × 0.8 m × 0.6 m. The water temperature was 20 ± 1 °C and the pH value was 7.5. The experimental groups were injected intraperitoneally with bacterial solutions of 10^3^, 10^4^, 10^5^, 10^6^, and 10^7^ colony-forming units (CFU)/g fish weight. The control group fish were injected with sterile PBS. After infection, the inoculated fish were observed for 10 consecutive days, and the number of deaths in each group was counted every day. Bacteria were isolated from diseased fish and identified again. The experiment was repeated three times. The 50% lethal dose (LD_50_) value of the H-701 strain was calculated by the method of Reed and Muench [22].

### 2.9. Drug Sensitivity

The isolated strain was tested for drug susceptibility using the Kirby-Bauer disk diffusion method [23]. In a biological safety cabinet (Esco, Singapore), strain H-701 was inoculated into the BHI liquid medium and cultured with shaking at 200 rotations per min at 28 °C for 20 h. The culture was centrifuged at 3000× *g* for 5 min to remove the liquid. The bacterial precipitation was adjusted to 0.5 McFarland standard for turbidity using sterile normal saline. The bacterial suspension was evenly spread on BHI agar plates (100 µL/plate), and paper discs containing antibiotics were then placed on each plate (Hangwei, Hangzhou, China). The plates were placed at a constant temperature of 28 °C for 24 h, and then the diameters of the inhibition zones were measured. The isolates were classified as sensitive (S), moderately sensitive (M), or resistant (R) according to the National Committee for Clinical Laboratory Standards (NCCLS).

### 2.10. Statistics

The data were analyzed by SPSS software (SPSS, version 19.0). Statistical analysis was performed using analysis of variance (ANOVA). The significance of differences between means was evaluated by Duncan’s multiple range test. Differences were considered significant at a level of *p*  <  0.05.

## 3. Results

### 3.1. Clinical Signs

**Figure 1:**
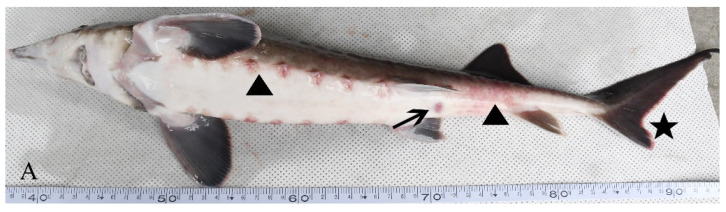

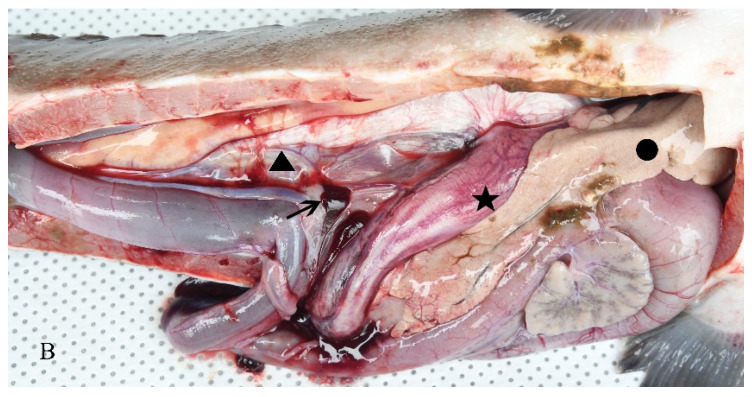
Clinical symptoms of an H-701-infected hybrid sturgeon: (**A**) Body surface (triangle), cloacal orifice (arrow), and fin (asterisk); (**B**) Ascites (triangle), liver whitening (dot), intestinal wall bleeding (asterisk), and spleen redness (arrow).

Diseased hybrid sturgeon floated on the water surface and rolled over as they could not keep their balance. Hemorrhages on the body surface and no feeding were observed. After dissection, the gill filaments were observed to be dark red. A small number of ascites were found in the abdominal cavity; the spleen was hyperemic, and the liver was white (Figure 1).

### 3.2. Colony Morphology

**Figure 2:**
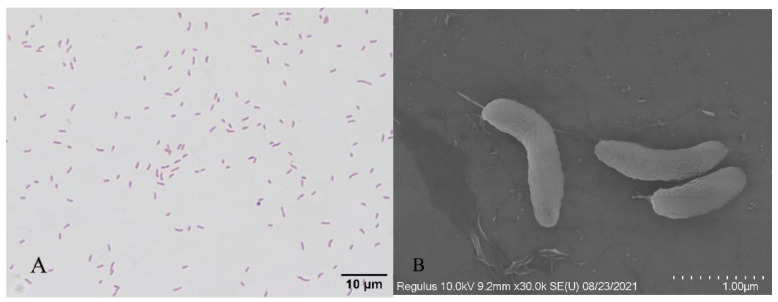
Gram staining and scanning electron microscopy of bacterium H-701: (**A**) Gram staining (bar scale: 10 µm); (**B**) Scanning electron microscopy (bar scale: 1 µm).

The H-701 strain was grown on a BHI agar medium, and the colony surface was smooth, moist, and grayish white. Optical microscopy showed that the cells were stained red (Gram-negative) after Gram staining and were curved, short, and rod-shaped (Figure 2). Scanning electron microscopy showed that the bacterial cells were arc-shaped and approximately 1.5 µm long (Figure 2).

### 3.3. Phylogenetic Analysis

**Figure 3:**
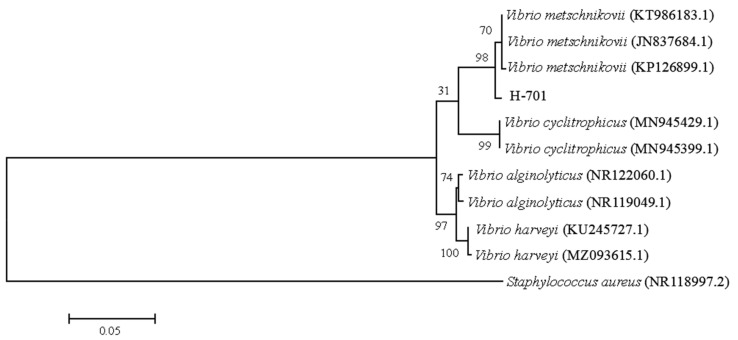
Phylogenetic tree of strain H-701 based on 16S rRNA gene sequences. The values at the node indicate the percentage of trees in which this grouping occurred after bootstrapping the data (1000 replicates). The scale bar indicates the number of substitutions per site.

The nucleotide sequence of the H-701 strain was compared with the NCBI database (http://blast.ncbi.nlm.nih.gov), and it showed >99.86% homology with the 16S rRNA gene sequence of *Vibrio metschnikovii* included in GenBank (GenBank accession no. KT986183.1). Using MEGA 6.0 software, the nucleotide sequences of the isolated strains were analyzed for homology with the nucleotide sequences of similar strains downloaded from NCBI. A phylogenetic tree was constructed using the neighbor-joining method [24]. The H-701 strain was clustered in the same clade as *V. metschnikovii* (Figure 3).

### 3.4. Bacterial Biochemical Identification

The Biolog microbial identification system compared the H-701 strain with the database on the basis of its biochemical reaction and determined that the strain was *V. metschnikovii* (Table 1).

### 3.5. Differential Leukocyte Counts

**Figure 4:**
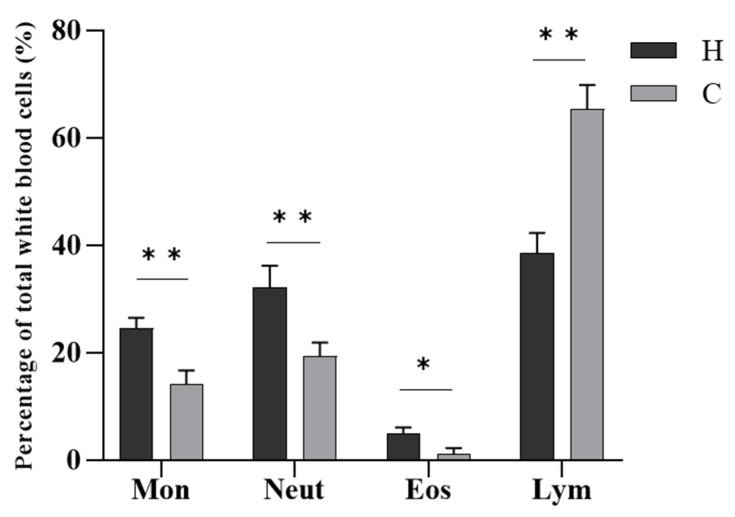
Differential leukocyte counts of hybrid sturgeon. Mon, monocytes; Neut, neutrophils; Eos, eosinophils; Lym, lymphocytes; H, diseased hybrid sturgeon (H-701); C, healthy hybrid sturgeon (Control); * *p* < 0.05; ** *p* < 0.01.

In terms of the number of white blood cells (Figure 4), the most numerous cells in diseased hybrid sturgeon were lymphocytes and neutrophils, followed by monocytes, and the least numerous were eosinophils. The most numerous white blood cells in healthy hybrid sturgeon were lymphocytes, followed by neutrophils and monocytes, and the least numerous were eosinophils. Diseased hybrid sturgeon had a significantly higher proportion of neutrophils (*p* < 0.01), monocytes (*p* < 0.01), and eosinophils (*p* < 0.05) and a significantly lower proportion of lymphocytes (*p* < 0.01) than healthy hybrid sturgeon.

### 3.6. Serum Biochemical Analysis

**Figure 5:**
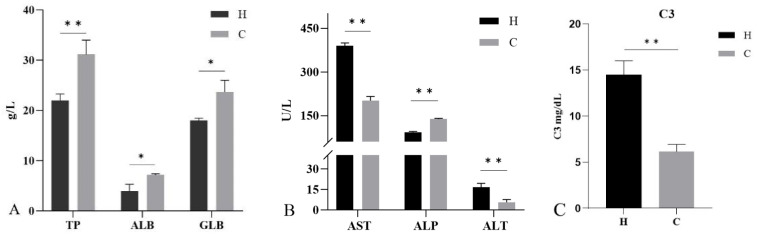
Serum biochemical indices of hybrid sturgeon: (**A**) Total protein (TP), albumin (ALB), and globulin (GLB); (**B**) Aspartate aminotransferase (AST), alkaline phosphatase (ALP), and alanine aminotransferase (ALT); (**C**) Complement C3 (C3). H, diseased hybrid sturgeon (H-701); C, healthy hybrid sturgeon (Control); * *p* < 0.05; ** *p* < 0.01.

The serum biochemical analysis showed (Figure 5) that the levels of TP, ALB, and GLB in diseased hybrid sturgeon were significantly lower (*p* < 0.05) than those in healthy hybrid sturgeon. In diseased hybrid sturgeon, the AST and ALT levels were significantly higher and ALP levels were significantly lower (*p* < 0.01) than those in healthy hybrid sturgeon. Complement C3 was significantly higher in diseased hybrid sturgeon than in healthy hybrid sturgeon (*p* < 0.01).

### 3.7. Histopathological Changes

**Figure 6:**
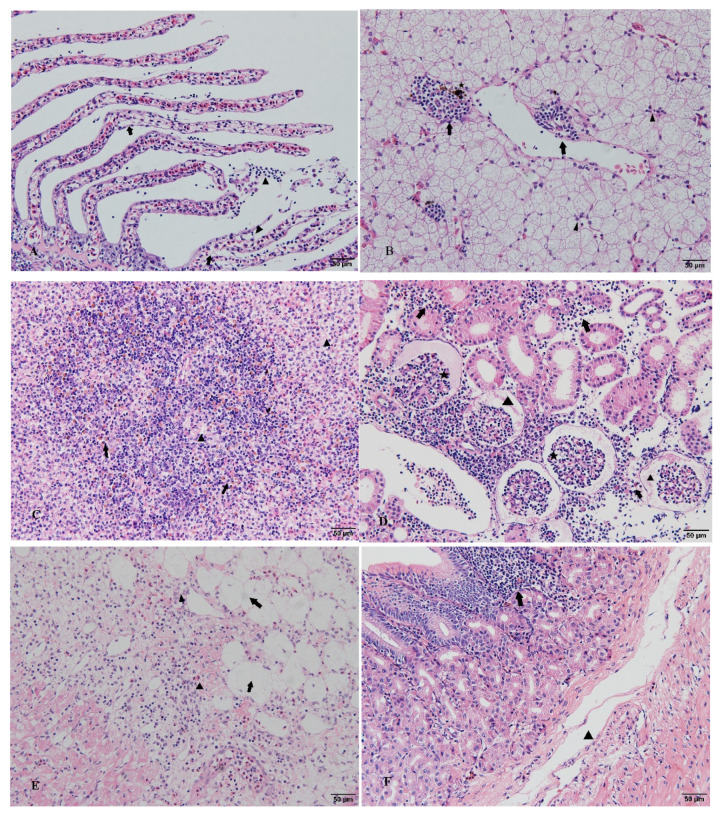
Histopathological observation of diseased hybrid sturgeon (scale: 50 µm): (**A**) Hyperplasia of epithelial cells in gill lamellae, hyperplastic fusion (arrow), inflammatory cell infiltration (triangle); (**B**) Hepatocyte swelling and degeneration, nuclear concentration and migration (triangle), and inflammatory cell infiltration (arrow); the presence of phagocytes in liver tissue (asterisk); (**C**) The white and red pulp structures of the spleen were blurred, cytopathic, and vacuolated (triangle) with numerous eosinophils (arrow); (**D**) Renal tubular epithelial cell necrosis, nuclear lysis, and inflammatory cell infiltration (lymphocytes, monocytes, eosinophils, etc.) of renal interstitial tissue (arrow). (**E**) Epicardial cyst, the proliferation of adipose tissue at the cyst site (arrow), nuclear deformation, dissolution, and extensive inflammatory cell infiltration (triangle). (**F**) The epidermal cells of the intestinal villi were exfoliated; the mucosal layer was infiltrated by inflammatory cells (arrow), the submucosa was loose, and the gap was enlarged (triangle).

The histopathological observations revealed pathological changes in several tissues of diseased hybrid sturgeon (Figure 6). The epithelial cells of the gill lamellae proliferated and formed a hyperplastic fusion. The liver showed pathological damage with hepatocytes enlarged, nuclei shifted, and inflammatory cells infiltrated. The spleen was cytopathic and vacuolized, and a large number of eosinophils appeared. Glomerular atrophy, renal tubular epithelial cell necrosis, nuclear lysis, and renal interstitial inflammatory cell infiltration also occurred. Moreover, epicardial cysts, adipose tissue hyperplasia, and nuclear deformation and dissolution were observed in diseased hybrid sturgeon. The epidermal cells of intestinal villi were exfoliated, and the submucosa was loose.

### 3.8. Pathogenicity

**Figure 7:**
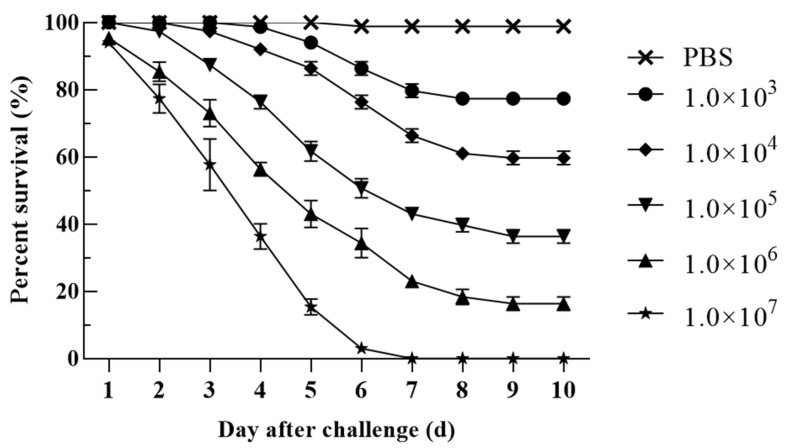
Pathogenicity of different concentrations of H-701 bacterial solution to hybrid sturgeon (Injection concentration: CFU/g fish weight).

In the pathogenicity test, death occurred in all fish groups treated with different concentrations of bacterial suspension, and none of the fish in the 10^7^ CFU/g fish weight experimental group survived (Figure 7). Dead hybrid sturgeon showed obvious bleeding on the body surface, redness and swelling of the anus, and visceral hyperemia. According to the experimental results, the LD_50_ of H-701 was 3.72 ± 0.929 × 10^4^ CFU/g fish weight. *V. metschnikovii* was again isolated from artificially infected hybrid sturgeon.

### 3.9. Drug Sensitivity Analysis

The H-701 strain isolated from diseased hybrid sturgeon was sensitive to the antibiotics florfenicol, enrofloxacin, doxycycline, ampicillin, penicillin, minocycline, and compound sulfamethoxazole; moderately sensitive to neomycin sulfate, gentamicin and amikacin; and resistant to polymyxin B (Table 2).

## 4. Discussion

*V. metschnikovii* mainly exists in the aquatic environment in nature [25]. As a zoonotic agent, it has been recognized as one of the pathogenic *Vibrio* species [26]. It mainly causes sepsis and diarrhea in humans and infection in aquatic animals [27,28,29]. *V. metschnikovii* produces hemolytic toxins and has often been isolated from seafood [30,31]. Exposure to wounds or consumption of contaminated seafood increases the risk of infection [31]. *Vibrio parahemolyticus* [32], *Vibrio mimicus* [33], and *Vibrio vulnificus* [34] have been reported in freshwater-cultured fish in China; however, to date, no infection of *V. metschnikovii* had been reported. In the present study, the pathogenic strain H-701 isolated from diseased hybrid sturgeon was identified as *V. metschnikovii*. The pathogenicity test showed that the LD_50_ was 3.72 ± 0.929 × 10^4^ CFU/g fish weight, and the H-701 strain had strong virulence to hybrid sturgeon. The symptoms of the artificially infected hybrid sturgeon were similar to those observed in naturally infected sturgeon. In addition, *V. metschnikovii* could be isolated from the artificially infected hybrid sturgeon again. These findings indicate that *V. metschnikovii* was the pathogenic bacterium that caused the disease in hybrid sturgeon.

In teleosts, white blood cells are key components of innate immune defense and are involved in the regulation of fish immune function [35]. The number of white blood cells in the blood is often used as a response to the health of fish [36]. White blood cells in sturgeon blood are divided into lymphocytes, granulocytes, and monocytes [36]. Among them, the proportion of lymphocytes was the highest, followed by neutrophils, with fewer eosinophils and monocytes [37]. Lymphocytes are an important component of the cellular immune response of the body, and a decrease in lymphocytes indicates poor immune function [38]. In the present study, the proportion of lymphocytes in the blood of diseased hybrid sturgeon decreased significantly, indicating a weakened immune system. Monocytes and granulocytes are the main phagocytes that kill pathogens chiefly by phagocytosis [39]. The proportion of monocytes, neutrophils, and eosinophils in the blood of hybrid sturgeon infected with *V. metschnikovii* was significantly increased, which enhanced phagocytosis. 

The serum biochemical indices of fish signify their health status, and crowding stress leads to significant changes in the blood physiological indices of hybrid sturgeon species [40]. The liver is an important site for protein synthesis in the fish body [41]. The levels of serum ALB and GLB are indicators of hepatocyte injury and immune response, respectively [42]. The serum ALB and GLB contents of diseased hybrid sturgeon were significantly reduced, indicating that hepatocytes were damaged and liver function decreased. ALT and AST exist in healthy hepatocytes, and when the hepatocytes are damaged, they escape the cells and enter the bloodstream [42,43]. In diseased hybrid sturgeon, the serum levels of ALT and AST increased, whereas the serum levels of ALP decreased, which is similar to the results in diseased *Pseudosciaena crocea* [44]. Complement C3 is an integral part of the innate immune system and has a role in protecting the host from invasion by foreign pathogens [45]. The increased content of complement C3 in the diseased hybrid sturgeon indicates the aggravation of the body’s inflammatory response.

Histopathological observation is an important method for fish pathology research [46,47]. Various pathological changes occurred in the tissues of hybrid sturgeon infected with *V. metschnikovii*. Among them, liver, kidney, and spleen tissue damage were the most serious, including cytopathic changes, nuclear lysis, and infiltration of a large number of inflammatory cells. Histopathological changes may be caused by bacterial toxin-induced cytopathic effects. Similar histopathological changes have been observed in fish infected with different *Vibrio* species [33,34,48]. 

Drug sensitivity tests can provide a basis for the identification of candidate drugs for the effective treatment of disease [49]. The H-701 strain isolated from diseased hybrid sturgeon was sensitive to various antibiotics such as florfenicol, enrofloxacin, doxycycline, and compound sulfamethoxazole. The test results were similar to those reported by Wei et al., but H-701 showed stronger sensitivity to penicillin and compound sulfamethoxazole [50]. This may be related to different origin strains or different resistance to antibiotics. Therefore, in the treatment of bacterial diseases, highly sensitive drugs should be selected according to the results of drug susceptibility tests for pathogenic bacteria.

This study confirmed that *V. metschnikovii* is the pathogen causing the disease of hybrid sturgeon. Whether *V. metschnikovii* is pathogenic to other freshwater fish and infectious in freshwater culture needs to be further verified. The differences in virulence genes between *V. metschnikovii* and other *Vibrio* that cause fish diseases need to be further studied.

## 5. Conclusions

In this study, a *V. metschnikovii* strain was isolated and identified from diseased hybrid sturgeon. A virulence test showed that it was pathogenic to sturgeon, with an LD_50_ of 3.72 ± 0.929 × 10^4^ CFU/g fish weight. The diseased hybrid sturgeon showed serious histopathological changes and significant changes in hematological parameters. The strain was sensitive to florfenicol, enrofloxacin, doxycycline, ampicillin, penicillin, minocycline, and compound sulfamethoxazole. In conclusion, *V. metschnikovii* was the pathogenic bacterium that caused a large number of deaths in hybrid sturgeon; the study highlights its potential risk in hybrid sturgeon aquaculture.

## Figures and Tables

**Table 1:** Biochemical identification results of strain H-701.

Reagent	Result *	Reagent	Result *
A1 Negative Control	N	E1 Gelatin	P
A2 Dextrin	P	E2 Glycyl-L-Proline	B
A3 D-Maltose	P	E3 L-Alanine	P
A4 D-Trehalose	P	E4 L-Arginine	B
A5 D-Cellobiose	B	E5 L-Aspartic Acid	P
A6 Gentiobiose	B	E6 L-Glutamic Acid	P
A7 Sucrose	P	E7 L-Histidine	P
A8 D-Turanose	B	E8 L-Pyroglutamic Acid	B
A9 Stachyose	N	E9 L-Serine	P
A10 Positive Control	P	E10 Lincomycin	B
A11 PH6	P	E11 Guanidine HCl	P
A12 PH5	N	E12 Niaproof 4	B
B1 D-Raffinose	B	F1 Pectin	P
B2 α-D-Lactose	N	F2 D-Galacturonic Acid	B
B3 D-Melibiose	B	F3 L-Galactonic Acid Lactone	B
B4 β-Methyl-D-Glucoside	P	F4 D-Gluconic Acid	P
B5 D-Salicin	B	F5 D-Glucuronic Acid	B
B6 N-Acetyl-D-Glucosamine	P	F6 Glucuronamide	B
B7 N-Acetyl-β-D-Mannosamine	B	F7 Mucic Acid	B
B8 N-Acetyl-D-Galactosamine	N	F8 Quinic Acid	B
B9 N-Acetyl Neuraminic Acid	N	F9 D-Saccharic Acid	B
B10 1% NaCl	P	F10 Vancomycin	P
B11 4% NaCl	P	F11 Tetrazolium Violet	N
B12 8% NaCl	B	F12 Tetrazolium Blue	P
C1 α-D-Glucose	P	G1 P-Hydroxy-Phenylacetic Acid	N
C2 D-Mannose	N	G2 Methyl Pyruvate	P
C3 D-Fructose	P	G3 D-Lactic Acid Methyl Ester	P
C4 D-Galactose	N	G4 L-Lactic Acid	P
C5 3-Methyl Glucose	B	G5 Citric Acid	P
C6 D-Fucose	B	G6 6α-Keto-Glutaric Acid	B
C7 L-Fucose	N	G7 D-Malic Acid	N
C8 L-Rhamnose	B	G8 L-Malic Acid	P
C9 Inosine	P	G9 Bromo-Succinic Acid	P
C10 1% Sodium Lactate	B	G10 Nalidixic Acid	N
C11 Fusidic Acida	B	G11 Lithium Chloride	B
C12 D-Serine	P	G12 Potassium Tellurite	P
D1 D-Sorbitol	P	H1 Tween 40	P
D2 D-Mannitol	P	H2 γ-Amino-Butyric Acid	B
D3 D-Arabitol	B	H3 α-Hydroxy-Butyric Acid	P
D4 Myo-lnositol	N	H4 β-Hydroxy-D, L-Butyric Acid	N
D5 Glycerol	P	H5 α-Keto-Butyric Acid	P
D6 D-Glucose-6-PO4	B	H6 Acetoacetic Acid	P
D7 D-Fructose-6-PO4	B	H7 Propionic Acid	N
D8 D-Aspartic Acid	N	H8 Acetic Acid	P
D9 D-Serine	B	H9 Formic Acid	N
D10 Troleandomycin	B	H10 Aztreonam	P
D11 Rifamycin SV	P	H11 Sodium Butyrate	P
D12 Minocycline	B	H12 Sodium Bromate	N

* Abbreviations: P, positive; N, negative; B, borderline; L, less than A1 well.

**Table 2:** Detection of drug sensitivity of the H-701 strain.

Medicine Name	Content	Inhibition Zone (mm)	Sensitivity *
Florfenicol	30 μg	22	S
Enrofloxacin	10 μg	27	S
Neomycin sulfate	30 μg	11	I
Doxycycline	30 μg	16	S
Ampicillin	10 μg	17	S
Penicillin	10 U	16	S
Gentamicin	10 μg	14	I
Minocycline	30 μg	17	S
Compound Sulfamethoxazole	23.75 μg	18	S
Tetracyclines	30 μg	22	S
Amikacin	30 μg	13	I
Polymyxin B	300 IU	9	R

* Abbreviations: S, susceptible; I, intermediate; R, resistant.

## Data Availability

The data presented in this study are available on request from the corresponding author.
